# Bactericidal effect of a diode laser on *Enterococcus faecalis* in human primary teeth—an in vitro study

**DOI:** 10.1186/s12903-018-0611-6

**Published:** 2018-08-31

**Authors:** Shanshan Dai, Gang Xiao, Ning Dong, Fei Liu, Shuyang He, Qingyu Guo

**Affiliations:** 1 Key Laboratory of Shaanxi Province for Craniofacial Precision Medicine Research, Xi’an, 710004 China; 2 Department of Pediatric Dentistry, Hospital of Stomatology Xi’an Jiaotong University College of Medicine, Xi’an, 710004 Shaanxi China

**Keywords:** Diode laser, Primary teeth, Disinfection, *Enterococcus faecalis*

## Abstract

**Background:**

In recent years, the diode laser (810 nm) has been used for root canal disinfection, which plays an important role in endodontic therapy. This study was undertaken to evaluate the disinfecting ability of a diode laser in experimentally infected root canals of primary teeth.

**Methods:**

Human retained mandibular primary anterior teeth without apical foramen resorption were selected and contaminated with *Enterococcus faecalis* for 21 days. The specimens were randomly divided into four groups: the negative group (no treatment), positive group (5.25% NaOCl), diode laser group (diode laser), and diode-NaOCl group (diode laser combined with NaOCl). The disinfecting abilities of the treatments were measured by the numbers of bacteria, scanning electron microscopy and confocal laser microscopy (live-dead staining).

**Results:**

Eighty teeth were selected. After irradiation and irrigation, the elimination of bacteria and the smear layer in the laser groups and positive group were significantly superior, compared with the negative group (*p* < 0.01). In the diode-NaOCl group, bacterial reduction reached nearly 100% on the surfaces of root canals; live bacteria were rarely observed, even in deeper dentinal tubules.

**Conclusion:**

Use of a diode laser, especially in combination with NaOCl, was effective for disinfecting infected root canals of primary teeth.

## Background

Dental caries and pulpitis are common diseases in children; notably, if the pulpitis cannot be controlled, it can result in primary tooth loss at early ages, affecting chewing, pronunciation, and aesthetics. Root canal therapy of the primary teeth is a commonly used therapeutic method. The main objective of endodontic treatment is to effectively eliminate bacteria and necrotic pulp tissue remnants from the root canal system [1], in order to preserve the teeth. Although most bacterial species are eliminated, some microorganisms may remain viable even after mechanical instrumentation, which may lead to an unfavorable root canal treatment outcome [2]. In addition, a biomechanical preparation cannot completely eliminate the microorganisms present in the root canal system; each technique has unique limitations [3]. Therefore, root canal system disinfection plays an important role in the success of endodontic treatment [4].

Numerous studies have proven that the bactericidal effect of a diode laser (810 nm) is based on thermal properties; furthermore, bacteria cannot develop resistance to laser exposure [5, 6]. A diode laser has been used in several areas of dentistry with promising disinfection outcomes [7–9]. Studies on the efficacy of lasers in endodontic therapy have mostly focused on permanent teeth [10], while studies on primary teeth have been rarely reported. Meanwhile, because of the complicated anatomical structure of the pulpal chamber [11], choosing the most effective disinfection protocol for pulp-infected primary teeth becomes particularly important.

This study evaluated the bactericidal efficacy of 810-nm diode laser irradiation and the combination of diode laser irradiation with 5.25% NaOCl in primary teeth by using scanning electron microscopy (SEM), confocal laser microscopy (CLM), and counting colony-forming units (CFUs).

## Methods

The protocol of this study was approved by the Research Ethics Committee of Xi’an Jiaotong University, Xi’an, China.

### Sample preparation

Retained human mandibular primary incisors without apical foramen resorption that had been extracted were included. The samples were stored temporarily in physiological saline solution at 4 °C. Teeth were decoronated and roots were normalized to 7 mm in length. The root canals were prepared using the crown down technique and stainless-steel K files up to size 30 (Dentsply Maillefer Ballaigues, Switzerland). Specimens were irrigated with 2 mL of 5.25% NaOCl during instrumentation. Teeth were ground longitudinally from the lingual portion with a high-speed diamond bur (MANI, Japan) to expose the inner parts of the root canals, followed by 2 mL of normal saline solution. A self-etching adhesive (3 M Dental Products, USA) was coated on the external surfaces of the roots to avoid microbial contamination. All sections were sterilized in an autoclave at 121 °C for 20 min. The root sections were then incubated in brain-heart infusion (BHI) broth for 24 h at 37 °C to isolate them from bacterial contamination.

### Female mold of the half side of the root canal preparation

In a randomly selected sample, the root canal was filled with molten red wax and solidified. A small amount of silicone rubber impression material was fully mixed and placed in plastic molding manufactured in-house (cylindrical, approximately 2.0 cm in diameter and 2.0 cm high). Then, the root was placed in the impression material until the material solidified; then, the root was removed. The female mold of the half side of the root canal was made and soaked in 1% peracetic acid for 30 min for disinfection after each sample was inserted into the mold.

### *Enterococcus faecalis* culture and inoculation

*Enterococcus faecalis* (ATCC 29212) was inoculated on BHI agar (Land Bridge, China) and incubated anaerobically at 37 °C for 24 h. A single colony was collected and aseptically resuspended in 10 mL of BHI broth. The sterilized specimens in 1 mL of sterile BHI broth were placed in 0.5 mL cultures (10^8^ CFU/mL as determined by a spectrophotometer). The specimens were then incubated under anaerobic conditions at 37 °C for 21 days. The medium was exchanged with fresh BHI broth every 2 days to remove dead bacteria and supply nutrition. All procedures were conducted under sterile conditions. After incubation, the specimens were removed from the tubes, rinsed in 2 mL of sterile saline, and then randomly divided into four groups.

### Experimental groups

All infected samples were placed into the female mold created from the silicone rubber impression material.

In the negative group (*n* = 20), there was no treatment.

In the positive group (*n* = 20), the sections were irrigated with 5 mL of 5.25% NaOCl for 60 s.

In the diode laser group (*n* = 20), the specimens were dried and irradiated with the diode laser at an output power of 2.0 W for 5 s and a wavelength of 810 nm in continuous mode (Lambda Dental Laser, LAMBDA Scientifica S.p.A, Italy). An optical fiber 200 μm in diameter was inserted into the root canal 1 mm short of the working length. The irradiation was repeated four times at 10-s intervals.

In the diode-NaOCl group (*n* = 20), the irradiation procedure was the same as in the diode laser group and was repeated four times. During each irradiation, the canals were irrigated with 1.25 mL of 5.25% NaOCl. All samples were rinsed with 2 mL of sterile saline to remove the remaining bacteria and NaOCl.

### Bacteriological evaluation

After the disinfection procedures, 10 samples from each group were subjected to CFU-counting evaluations. Five samples among them were split into three equal pieces (namely the coronal, middle, and apical regions of the root canal). Each specimen was placed into a sterile Eppendorf tube with 1 mL of physiological saline solution and sonicated at 7 W for 60 s using an ultrasonic device [12] (P5 Newtron XS, Satelec, France). After mixing, the liquid was diluted in log base 10 steps and 100 μL of each dilution was inoculated onto BHI agar plates, which were then incubated for 24 h at 37 °C under anaerobic conditions. Bacterial CFUs were observed and counted.

### Examination by SEM

Another five samples from each group were fixed in 2.5% glutaraldehyde for 24 h. Following dehydration in graded concentrations of ethanol, the specimens were air dried, coated with a layer of platinum (Ion Sputter E-1045, Hitachi, Japan), and observed by SEM (S-4800, Hitachi, Japan).

The coronal, middle, and apical regions were examined. Photographs were taken at various magnifications ranging from 30× to 500,000× by the same operator. Two observers blinded to the group allocations evaluated the remaining smear layers based on a scoring system described by Takeda [13]. A score of 1 represents no smear layer or debris evident in the dentinal tubules; a score of 2 represents a few regions of dentinal tubules covered with a smear layer and debris, with most tubules cleaned and opened; a score of 3 represents that most regions of dentinal tubules were covered with a smear layer and debris with a few tubules cleaned and opened; and a score of 4 represents dentinal tubules completely covered with a smear layer and debris.

### Analysis of CLM

The remaining samples were stained with a LIVE/DEAD BacLight Bacterial Viability Kit (L7012, Life, USA) for 15 min, in accordance with the manufacturer^’^s instructions. The stained samples were cut into three equal parts using a low-speed diamond saw (SYJ150, MTI, USA). The specimens were covered with aluminum foil to prevent light exposure and maintained at 4 °C. The samples were then mounted onto glass slides and visualized under an Olympus confocal laser scanning microscope (FV10-ASW, Olympus, Japan) at 20× magnification. Green and red fluorescence were detected using wavelengths of 488 nm and 543 nm, respectively. The digital images were imported into the Image-Pro Plus 6.0 program (MediaCybernetics, USA) to evaluate the efficacy of disinfection by measuring the green-to-red fluorescence area ratio in each portion.

### Statistical analysis

The statistical analysis was performed with SPSS 19.0 for Windows (SPSS, China). An analysis of variance (ANOVA) model was used to compare the mean CFUs and fluorescence area ratios among the groups. The smear layer scores were analyzed using the Kruskal–Wallis test to estimate the ultrastructural morphological changes. The level of significance was set at α = 0.05.

## Results

Eighty teeth were selected and randomized into the four groups equally (*n* = 20 per group).

### Bacteriological evaluation

The numbers of recovered bacteria from different parts of specimens from each group are presented in Table 1. The negative group demonstrated the least bactericidal effect, followed by the positive group, diode laser group, and diode-NaOCl group. In the negative group, 10^5^ CFU/mL of bacteria were detected. After irrigation and irradiation, bacterial reductions were significantly greater on the surfaces of the root canals (*p* < 0.01). Disinfection of the root canal portions in the diode laser group was superior to that in the positive group except for the apical part (*p* < 0.05). In addition, the diode-NaOCl group showed that the bacterial reduction reached nearly 100% on the surface of each part of the root canal and there were significant differences between each of the group pairs (*p* < 0.01). In each group, the bacteria were eliminated more effectively in the coronal and middle parts compared with the apical parts (*p* < 0.05).

**Table 1:** Bacterial counts (CFU/mL, mean ± SD) and bacterial reductions (BR) in the different groups

	negative group	positive group		diode laser group		diode-NaOCl group	
	Mean ± SD (log10)	Mean ± SD (log10)	RD (%)	Mean ± SD (log10)	RD (%)	Mean ± SD (log10)	RD (%)
Coronal region	5.82 ± 4.74^A^	4.51 ± 3.78^B a^	95.12	4.38 ± 3.60^C a^	99.44	3.30 ± 1.78^D a^	99.74
Middle region	5.72 ± 4.83^A^	4.49 ± 3.70^B a^	94.13	4.36 ± 3.78^C a^	95.70	3.30 ± 2.70^D a^	99.70
Apical region	5.70 ± 4.80^A^	4.51 ± 3.85^B b^	93.25	4.40 ± 3.78^B b^	94.94	3.30 ± 2.70^C b^	99.54
Integrity	5.82 ± 4.15^A^	4.58 ± 3.90^B^	94.16	4.36 ± 3.85^C^	96.47	3.30 ± 2.78^D^	99.72

SD, standard deviation; BR, bacterial reduction; CFU, colony-forming units

Data with different uppercase letters (A, B, C, D) indicate significant differences within each group (*p* < 0.05)

Data with different lowercase letters (a, b) indicate a significant difference within each region (*p* < 0.05)

### SEM examination

**Fig. 1:**
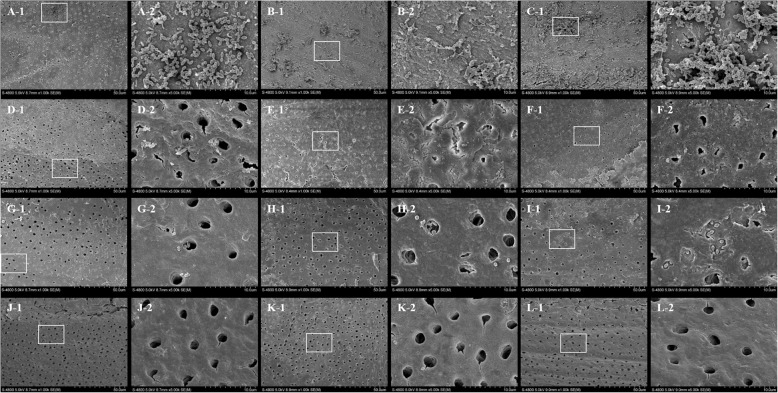
Scanning electron microscopy (SEM) observation of the smear layer and *Enterococcus faecalis* removed after treatment in each group. Negative group: a heavy smear layer and biofilm-like structures are observed on the surface of the root canal (A–C, A: coronal region; B: middle region; C: apical region). Positive group: a few smear layer-covered areas and tubules are visible but have not completely opened and the remaining biofilm is shrunken (D–F, D: coronal region; E: middle region; F: apical region). Diode laser group: a small amount of smear layer is evidenced on the smooth root canal surface and sparse bacteria are detected (G–I, G: coronal region; H: middle region; I: apical region). Diode-NaOCl group: the smear layer is uniformly clean and the tubules are opened; there are almost no bacteria on the root canal surface (J–L, J: coronal region; K: middle region; L: apical region). Magnification: A~L-1 1000×; A~L-2 5000×

The kappa value showed excellent reliability and reproducibility between the two observers at 0.89. The calculated smear layer scores from different parts of the specimens from each group are presented in Table 2. A heavy, continuous smear layer was observed on the entirety of the root canal walls in the negative group and the debris and bacteria displayed a smooth surface (Fig. 1A–C). In the positive group, a few shrunken smear layers were discovered and the dentinal tubules were visible but not completely opened (Fig. 1D–F). In the diode laser group, few smear layers were observed and sparse bacteria were detected (Fig. 1G, H) except in the apical part (Fig. 1I). The diode-NaOCl group presented the best disinfection outcome. The smear layer was thoroughly cleaned, the tubules were opened, and almost no bacteria existed on the root canal surface (Fig. 1J–L). The analysis showed that the combination of diode laser irradiation and NaOCl resulted in the lowest score (*p* < 0.01), followed by the diode laser group, positive group, and negative group (*p* < 0.05). Meanwhile, in all experimental groups except for the diode-NaOCl group, the smear layers on the coronal and middle regions were removed more effectively than those on the apical regions (*p* < 0.05).

**Table 2:** Smear layer scores (Mean ± SD) in different groups and three regions of root canal

	negative group	positive group	diode laser group	diode-group
Coronal region	3.71 ± 0.46^A^	2.57 ± 0.49^B a^	1.86 ± 0.35^C a^	1.14 ± 0.35^D a^
Middle region	3.86 ± 0.35^A^	2.43 ± 0.49^B a^	1.71 ± 0.42^C a^	1.14 ± 0.35^D a^
Apical region	3.71 ± 0.45^A^	3.14 ± 0.35^B b^	2.43 ± 0.49^B b^	1.29 ± 0.45^D a^

Data with different uppercase letters (A, B, C, D) indicate significant differences within each group (*p* < 0.05)

Data with different lowercase letters (a, b) indicate significant difference within each region (*p* < 0.05)

### CLM analysis

**Fig. 2:**
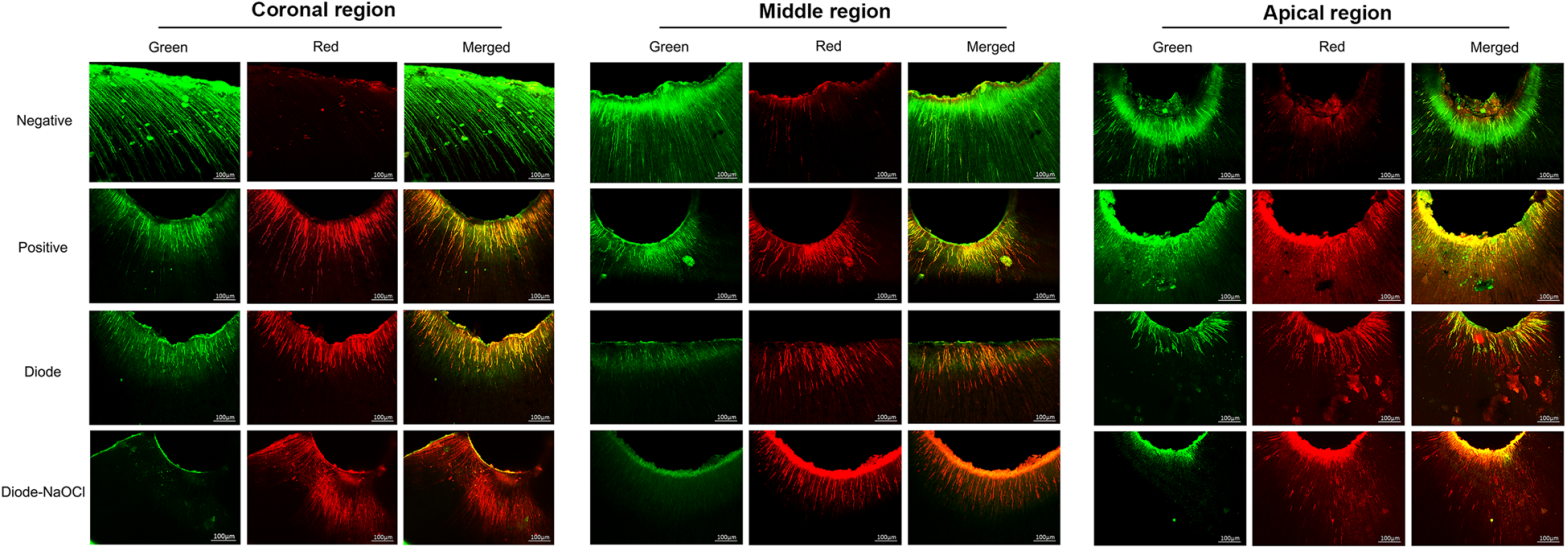
Confocal laser microscopy analysis of the distributions of vital and non-vital bacteria in each region

CLM analysis allowed us to distinguish viable from nonviable bacteria on root canal walls and in dentinal tubules. To detect the presence of green bacteria (vital) in the biofilms or red bacteria (non-vital) in the biofilms, the means and standard deviations of the green-to-red fluorescence area ratios were determined for each fragment and are shown in Table 3. From high to low, the area ratios in the groups were as follows: the negative group, positive group, diode laser group, and diode-NaOCl group. In the positive group, the ratio was close to 1.0 except in the apical region where it was greater than 1.0 (*p* > 0.01). In the diode laser group and diode-NaOCl group, the area ratios in each portion were less than 1.0 (*p* > 0.01). The corresponding images are shown in Fig. 2. Regardless of the root portion, the negative group revealed a much greater amount of green (vital) fluorescence than other groups (*p* < 0.01). Conversely, the rest of the groups presented various degrees of green and red (non-vital) fluorescence. In the positive group, the amounts of green and red fluorescence were equal, which showed statistically significant differences with the other groups (*p* < 0.01). The diode laser group showed much more red fluorescence in the dentinal tubules indicative of non-vital bacteria in the biofilms and the merged picture was dominated by red fluorescence (*p* < 0.01). Moreover, the diode-NaOCl group showed little green and mostly red fluorescence and no green fluorescence in the deeper dentinal tubules (*p* < 0.01).

**Table 3:** The ratio between green and red fluorescence area (Mean ± SD) in different groups

	negative group	positive group	diode laser group	diode-NaOCl group
Coronal region	37.15 ± 2.35^a^	0.93 ± 0.02^b^	0.62 ± 0.01^c^	0.02 ± 0.003^d^
Middle region	14.81 ± 2.73^a^	0.96 ± 0.02^b^	0.64 ± 0.04^c^	0.05 ± 0.004^d^
Apical region	10.98 ± 1.38^a^	1.67 ± 0.04^b^	0.47 ± 0.02^c^	0.12 ± 0.003^d^

Data with different lowercase letters (a, b, c, d) indicate a significant difference within each group (*p* < 0.01)

## Discussion

Disinfection is one of the key steps in successful root canal treatment. The methods include microbial reduction through mechanical preparation removal of residual pulp tissue and debris, and chemical preparation to maintain teeth. Once tooth reserve failure, it would affects children’s chewing, aesthetics, and eruption of permanent teeth.

*Enterococcus faecalis* is a gram-positive facultative anaerobic organism that can be commonly isolated from failed root canal treatment [14]. Several studies showed that *E. faecalis* bacteria became more resistant in the starvation phase and were difficult to eradicate [14–16]. Additionally, conventional irrigations cannot completely eliminate it. The results from our bacteriological evaluation showed that the diode-NaOCl group demonstrated the lowest amount of bacteria, followed by the diode laser group and positive group. In the diode-NaOCl group, there was a reduction of at least 99.7% in *E. faecalis* on the surface of the root canal. This finding could be attributed to the efficient removal of the smear layer from the dentinal surface by the diode laser, which resulted in improved contact between the NaOCl and bacteria. This finding makes the results of this study noteworthy. Hence, it could be concluded that the bactericidal and permeation effects of NaOCl, which are well known, are enhanced by the use of a diode laser.

In our experiment, the sample was divided into two parts; however, this is difficult with primary teeth [17]. Therefore, we first used a silicone rubber impression material to prepare a mold of the root. The mold could simulate the state of rinse reflux in the apical portion, which provided a better simulation of the root. In comparison with other parts of root canal, removal of the smear layer and elimination of bacteria were significantly greater in the coronal and middle regions than in the apical region. Our results suggested that most residual bacteria are in the apical region following disinfection; this is why we focus on root canal filling in the apical portion [18]. These findings may be due to the structure of the apical portion of the root, which does not facilitate irrigation, and the tip of the laser, which cannot reach the apical portion. However, in the diode-NaOCl group, there were no statistically significant differences among the three regions. The area ratio of the green-to-red fluorescence was also the lowest among the groups, which suggested the most effective disinfection from the lack of live bacteria. This again proved that a diode laser in combination with NaOCl had greater disinfectant properties in the apical portion.

The diode laser technique has been performed in endodontics treatment for a decade [19]. It is well known that different parameters of lasers and fibers, the output power of the laser, and the duration of application have different effects on disinfection [6]. The thermal effect is the most important point to be considered in laser applications. A temperature rise to a critical level could have deleterious effects on the tissues surrounding the tooth. The temperature increases by approximately 10 °C and a treatment duration of 1 min can cause irreversible injury to periodontal tissues [20]. Gutknecht et al. [21] demonstrated that diode laser irradiation for 5 s, with 10 s of resting time, should be considered to avoid a temperature rise to an undesired level. Schoop et al. [22] also drew similar conclusions that a diode laser showed the lowest temperature increases compared with other laser devices and was suitable for the disinfection of root canals. Therefore, according to the information, we adopt a safe mode of diode laser to perform root canal therapy. Several studies have shown that a 3.0 W diode laser with an 810-nm wavelength could eliminate bacteria when used on permanent teeth [5]. Because of the different structures and components between permanent and primary teeth, the laser power used for permanent teeth may not be suitable for primary teeth [23]. Our pilot experiment revealed that a 2.0 W diode laser was the most efficient at removing the smear layer on primary teeth while avoiding dentinal melting [24]. In our study, diode laser irradiation was performed with an output of 2.0 W for 5 s followed a 10-s interval or irrigation, in accordance with the method used in the study by Akyuz [25]. However, further studies are required to confirm the biological safety of diode laser application.

## Conclusions

In conclusion, both diode laser groups, especially the diode-NaOCl group, showed satisfactory bactericidal effects in experimentally contaminated root canals of primary teeth. The combination of a diode laser and NaOCl could be an ideal protocol to improve the success rate of therapy and reduce negative impacts on children, and can provide helpful guidance for the clinical application of primary root canal disinfection.

## Abbreviations

ATCC: American Type Culture Collection
BHI: Brain Heart Infusion
CFUs: Colony-Forming Units
CLM: Confocal Laser Microscopy
E. faecalis: Enterococcus faecalis
NaOCl: Sodium hypochlorite
SEM: Scanning Electron Microscopy
